# Editorial: Microbial ecology supporting growth of free-living amoebae in natural and engineered water systems

**DOI:** 10.3389/fmicb.2025.1620877

**Published:** 2025-05-29

**Authors:** Elliott P. Barnhart, Isabel Douterelo, Matthew J. Morgan, Geoffrey J. Puzon

**Affiliations:** ^1^U.S. Geological Survey, Wyoming-Montana Water Science Center, Helena, MT, United States; ^2^School of Mechanical, Aerospace and Civil Engineering, University of Sheffield, Sheffield, United Kingdom; ^3^CSIRO Environment, Canberra, ACT, Australia; ^4^CSIRO Environment, Waterford, WA, Australia

**Keywords:** *Naegleria fowleri*, amoeba, water, pathogens, microbial ecology

Free-living amoebae (FLA) are widespread in natural and engineered aquatic environments and are increasingly recognized as key players in aquatic ecosystems and public health. While many FLAs are benign, species in several genera—including *Naegleria fowleri, Acanthamoeba* spp., and *Balamuthia mandrillaris*—are capable of causing severe human infections such as primary amoebic meningoencephalitis, Acanthamoeba keratitis, and granulomatous amoebic encephalitis. Beyond their pathogenic potential, FLAs function as environmental reservoirs and vectors for opportunistic bacterial pathogens, such as *Legionella pneumophila, Mycobacterium intracellulare, Chlamydia*, and *Shigella spp*., supporting their survival, protection, and dissemination in diverse water systems.

Environmental changes are expanding the ecological niches of thermotolerant FLAs like *N. fowleri*, yet microbial interactions within FLA-associated ecosystems remain underexplored. Most research has traditionally focused on either FLAs or bacterial pathogens in isolation, neglecting the complex ecological relationships that influence FLA and bacterial presence, persistence, and virulence.

The aim of this Research Topic was to provide a comprehensive look into current knowledge of the microbial ecology of FLAs in water systems, with a particular focus on ecological, molecular, and environmental factors influencing their interactions and public health implications. This Research Topic brings together key studies that deepen our understanding of FLA behavior and their interactions with bacterial prey and human pathogens.

Fan et al. contributed a broad review titled “*Social life of free-living amoebae in aquatic environment—comprehensive insights into interactions of free-living amoebae with neighboring microorganisms*”. The authors synthesize evidence of how FLA interactions with bacteria, viruses, and other eukaryotes can shape their ecological roles and modulate their pathogenic potential. This study highlights the dual role of FLAs as environmental sentinels and facilitators of pathogen transmission, and their importance in both natural and engineered aquatic systems.

Goudot et al. investigated the material-dependent colonization patterns of *N. fowleri* in biofilms under thermophilic conditions typical of industrial cooling systems. Their study, “*Growth dynamic of biofilm-associated Naegleria fowleri in freshwater on various materials*”, revealed that while amoeba colonized most materials, brass surfaces significantly inhibited their growth—likely due to antimicrobial properties of copper. Additionally, bacterial prey abundance directly influenced amoeba proliferation, with maximum growth rates reaching 0.18 ± 0.07 h^−1^ and generation times around 4 h. These findings inform the impact of material selection strategies on FLA colonization in water infrastructure.

Environmental factors such as salinity and temperature were further explored by Arberas-Jiménez et al. in their study on *Acanthamoeba griffini* and *N. fowleri* growth dynamics using laboratory-based systems. The authors demonstrated that while both species are thermotolerant, *A. griffini* exhibited greater halotolerance, and neither species survived salinity levels equivalent to seawater. These insights indicate the potential for salinity-based mitigation in water treatment and recreational settings.

Expanding the focus to premise plumbing systems, Gomez-Alvarez et al. conducted a metagenomic analysis of opportunistic premise plumbing pathogens and their interactions with phagocytic amoebae in a simulated residential setting. The co-occurrence of *L. pneumophila* and *M. intracellulare* with *Vermamoeba vermiformis* during periods of water stagnation indicates that amoebae may act as protective hosts, shielding pathogens from disinfectants and promoting persistence within home plumbing systems. This study reinforces how understanding interspecies interactions can help account for both microbial ecology and infrastructure behavior when managing waterborne health risks.

Collectively, the articles in this Research Topic reveal a consistent theme: FLAs are not only resilient inhabitants of aquatic environments but also key players in the dynamics of the microbiome by shaping the survival, growth, and dissemination of waterborne pathogens. Their interactions with microbial communities and response to environmental stressors—such as material composition, temperature, stagnation, and salinity—have direct implications for water quality, infrastructure design, and public health.

In conclusion, this Research Topic highlights how integrative approaches that bridge microbiology, engineering, and environmental science can provide a more holistic perspective of multi-domain microbial dynamics. Understanding the ecological roles of FLAs and their interactions with other microbes can help design effective surveillance, risk mitigation, and infrastructure resilience strategies in the face of water management challenges and aging water infrastructure. We hope these studies will inspire future interdisciplinary research that continues to illuminate the microbial complexity of water systems and help safeguarding public health.

